# Biocompatibility and Biomechanical Effect of Single Wall Carbon Nanotubes Implanted in the Corneal Stroma: A Proof of Concept Investigation

**DOI:** 10.1155/2016/4041767

**Published:** 2016-12-27

**Authors:** Alfredo Vega-Estrada, Joaquin Silvestre-Albero, Alejandra E. Rodriguez, Francisco Rodriguez-Reinoso, Jose A. Gomez-Tejedor, Carmen M. Antolinos-Turpin, Laurent Bataille, Jorge L. Alio

**Affiliations:** ^1^Keratoconus Unit, Vissum Corporation, Alicante, Spain; ^2^Research and Development Department, Vissum Corporation, Alicante, Spain; ^3^Division of Ophthalmology, Universidad Miguel Hernández, Alicante, Spain; ^4^Departamento de Química Inorgánica, Universidad de Alicante, Alicante, Spain; ^5^Center for Biomaterials and Tissue Engineering, Universitat Politècnica de València, Valencia, Spain

## Abstract

Corneal ectatic disorders are characterized by a progressive weakening of the tissue due to biomechanical alterations of the corneal collagen fibers. Carbon nanostructures, mainly carbon nanotubes (CNTs) and graphene, are nanomaterials that offer extraordinary mechanical properties and are used to increase the rigidity of different materials and biomolecules such as collagen fibers. We conducted an experimental investigation where New Zealand rabbits were treated with a composition of CNTs suspended in balanced saline solution which was applied in the corneal tissue. Biocompatibility of the composition was assessed by means of histopathology analysis and mechanical properties by stress-strain measurements. Histopathology samples stained with blue Alcian showed that there were no fibrous scaring and no alterations in the mucopolysaccharides of the stroma. It also showed that there were no signs of active inflammation. These were confirmed when Masson trichrome staining was performed. Biomechanical evaluation assessed by means of tensile test showed that there is a trend to obtain higher levels of rigidity in those corneas implanted with CNTs, although these changes are not statistically significant (*p* > 0.05). Implanting CNTs is biocompatible and safe procedure for the corneal stroma which can lead to an increase in the rigidity of the collagen fibers.

## 1. Introduction

Corneal debilitating disorders are characterized by progressive changes of the geometry of the tissue that leads to an irregular astigmatism that negatively impacts the patient's visual system [1]. There are basically two types of corneal ectatic disorders; the first one is comprised of those pathologies of primary origin, such as keratoconus and pellucid marginal degeneration, and the other kind of pathologies, that appear secondary to a corneal photoablative procedure, is known as Ectasia post-LASIK or Iatrogenic Keratectasia [2]. The underlying mechanism responsible for the corneal weakening that induces the aforementioned abnormalities is the biomechanical alterations of the collagen fibers within the corneal stroma [3–5]. Contact lens wearing, intracorneal ring segment implantation, thermokeratoplasty procedures, cornea transplant, and corneal collagen cross-linking have been described as treatment alternatives to manage such a pathological condition [6–11]. Until the date, riboflavin/UV light exposure corneal collagen cross-linking is the only treatment option that has proved to stop the progressive nature of the corneal ecstatic disorders [12]. However, it is a long and uncomfortable surgical procedure for both the patient and the surgeon and it is also a technique not exempt of complications [12].

Carbon nanostructures, mainly carbon nanotubes (CNTs) and graphene, have attracted great attention in the last few years due to their small size and their extraordinary physicochemical properties (they are the stiffest and strongest materials known). Whereas graphene is a flat monolayer of carbon atoms tightly packed into a two-dimensional (2D) honeycomb lattice, carbon nanotubes can be visualized as rolled sheets of graphene built from sp2-carbon units. Previous studies described in the literature have shown that carbon nanomaterials (mainly CNTs) offer potential structural reinforcement in hydrogels to be used in regenerative medicine due to their high mechanical strength and good biocompatibility [13–15]. Furthermore, CNTs have shown promising biochemical properties, such as strong cell adhesion in multiwall carbon nanotube-coated collagen sponge and protein absorption [16–18].

The purpose of the present investigation was to assess if carbon nanomaterials are safe and compatible with the ocular tissues. We also aimed to evaluate the mechanical properties of the corneal stroma after implantation of the CNTs. To the best of our knowledge, this is the first time that carbon nanomaterials influence on corneal rigidity is reported and also the first to evaluate the corneal tolerance of such nanostructures.

## 2. Materials and Methods

### 2.1. Experimental Animals

White New Zealand rabbits were used in this study as reference animal models (internationally homologated) for the safety evaluation of new ocular concepts. Animal care and treatment procedures were in accordance with the standards of the EU Directive 2010/63/EU for animal experiments, in conformity to National Guidelines for animal usage in research and with the regular rules (Real Decreto 53/2013) of the Animal Experimentation Service of the Miguel Hernandez University, Alicante, Spain and after the Ethics Experimental Animal Committee approval.

**Figure 1:**
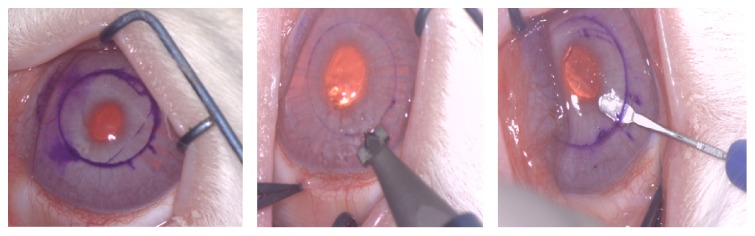
Rabbit eye at the moment of the surgical procedure showing the different steps for the creation of the pocket in the corneal stroma.

**Figure 2:**
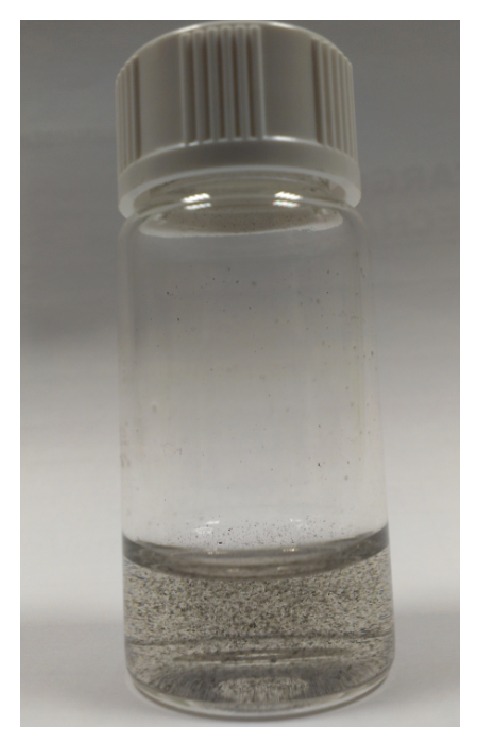
Appearance of the dispersion composed of carbon nanotubes (CNTs) mixed with saline solution in which concentration is 1 mg/mL.

Twenty-four eyes of white New Zealand rabbits were included in this study. The animals were anesthetized with subcutaneous injections of a mixture of ketamine hydrochloride 20 mg/kg (Imalgene 1000; Merial, Lyon, France) and xylazine hydrochloride 4 mg/kg (Xilagesic 2%; Laboratorios Calier, Barcelona, Spain), and ocular topic double anesthetic (tetracaine 0.1% and oxibuprocaine 0.4%, Colircusi; Alcon Cusi SA, Barcelona, Spain). The study was designed based on four experimental animal groups as follows: Group 1 (Control group): 5 eyes in which no surgical procedure was performed; Group 2 (Pocket group): 5 eyes in which a space (pocket) was created in the middle of the corneal stroma. In order to create the pocket, we first marked a 6 mm diameter circle on the surface of the cornea. Then a vertical incision of 300 microns in depth was performed using a calibrated diamond knife. Finally, the dissection of the corneal stroma through an area of 6 mm and at 300 microns in depth was performed using a minicrescent surgical knife (Figure 1). Group 3 (Reference 1 group) was 7 eyes in which a pocket was created and a dispersion composed of carbon nanotubes (CNTs) mixed with saline solution was injected inside the pocket. The concentration of this composition was 0.1 mg/mL. Group 4 (Reference 2 group) was 7 eyes in which a pocket was created and a dispersion composed of CNTs mixed with saline solution was injected inside the pocket. The concentration of this composition was 1 mg/mL (Figure 2).

Antibiotic prophylaxis was topically applied using Tobramycin/Dexamethasone (Tobradex, Alcon) and Chloramphenicol ointment (Oftalmolosa Cusi, Alcon) twice a day for 7 days. Subcutaneous Buprenorphine (0.05 mg/kg) and Paracetamol in drinking water (100 mg/mL) were also used as analgesic for the first days after surgery.

After the injection of the composition containing CNTs, the rabbits were kept under observation for a period of 3 months.

Afterwards, euthanasia was performed in rabbits and a corneoscleral rim of approximately 15 mm in diameter was dissected for histopathology and biomechanical evaluations.

### 2.2. Preparation of Carbon Nanotubes

Single wall carbon nanotubes (SWCNTs) were used in the current experimental protocol. SWCNTs were prepared by laser ablation of a graphite rod in the presence of Cobalt (Co) and Nickel (Ni) according to previous recipes reported in the literature [19, 20]. Synthesized SWCNTs were purified using 15% hydrogen peroxide solution under reflux at 100°C for 5 hours to remove the amorphous carbons. The residual catalysts of Ni and Co were removed by 1 M hydrogen chloride solution, followed by a washing step using double distilled water. Synthesized SWCNTs were dissolved in physiological solution (buffer saline solution) before being incorporated in the cornea using a final concentration of 0.1 mg/mL and 1 mg/mL (see Figure 2).

### 2.3. Histopathology Evaluation

After euthanasia, a piece of the corneal bottoms was dissected and fixed in 10% phosphate buffered formaldehyde until tissue processing. The safety and biocompatibility of the CNTs dispersion composition in the corneal tissue were assessed by means of histopathology examination of the samples 3 months after the surgery. Histological sections were obtained with a thickness of 3 microns and were stained with Hematoxylin and Eosin, blue Alcian, and Masson's trichrome.

### 2.4. Biomechanical Evaluation

Mechanical properties of the cornea were analyzed by performing stress-strain measurements in order to determine the modulus of elasticity of the corneal tissue. For this purpose, we dissected a strip of corneoscleral tissue with a dimension of 5 × 15 mm that then was clamped into a Microtest SCM 3000 95 universal testing machine in tensile mode (Microtest SA, Madrid, Spain) with load cell of 15 N at room temperature. By means of strain control, samples underwent deformation in a ramp with a constant speed of 1 mm/min. The equipment registered the force that was necessary to perform this deformation, and then with the data that was obtained, stress/strain graphics were drawn. Young's modulus of the samples was obtained as the slope of the linear region in the stress-strain curve at approximately 10% strain. Yield strength was calculated as the stress at which this linear behaviour is finished; that is, it began to deform plastically. Failure strain was calculated at the point at which the sample started to break. Between five and seven measurements were performed for each group. Results are given as the average plus/minus standard deviation.

### 2.5. Statistical Analysis

The statistical analysis was carried out using the one-way analysis of variance (ANOVA) with R 3.1.0 software. The level of statistical significance was *p* < 0.05 in all cases.

## 3. Results

### 3.1. Clinical Evaluation

**Figure 3:**
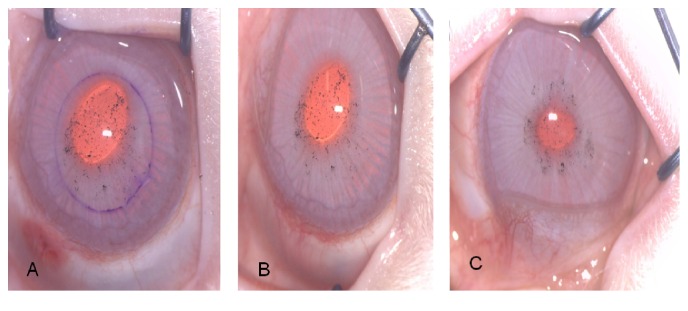
Same eye of a rabbit at the moment of the surgical procedure (A), 7 days after CNTs implantation (B) and at 3 months after the surgery (C).

The present investigation was performed by means of an experimental protocol where 24 eyes of New Zealand white rabbits were implanted with CNTs in the corneal stroma and were kept under observation during a period of 3 months.  Figure 3 corresponds to a series of images showing the rabbit's eyes at the moment of the surgical procedure, 7 days after CNTs implantation, and at 3 months during the last follow-up. At the moment of the surgery, the CNTs can be appreciated, using the surgical microscope under high magnification, as small black dots distributed along the previously performed corneal pocket. One week after the procedure, the corneal tissue is completely transparent, with no epithelial defect, without corneal edema and no signs of stromal or anterior chamber inflammation. At three months, the corneal tissue remains transparent, with the small black dots corresponding to the CNTs implanted and no clinical signs of ocular inflammation. There were no complications in any of the 14 eyes implanted with the CNTs and there was no need of an early euthanasia in any of the rabbits during the follow-up period.

### 3.2. Histopathology Evaluation

**Figure 4:**
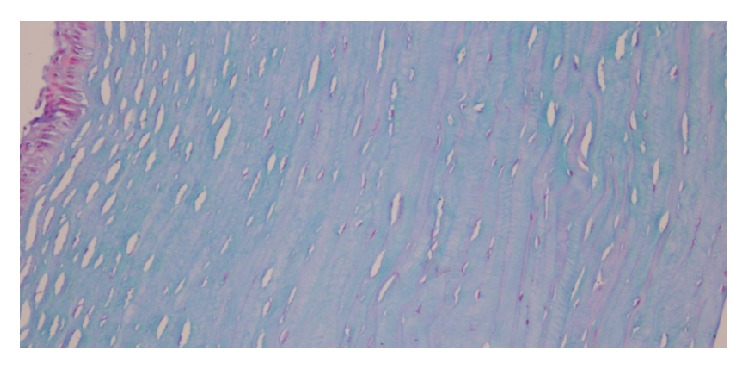
Corneal stroma sample in vertical orientation stained with blue Alcian.

**Figure 5:**
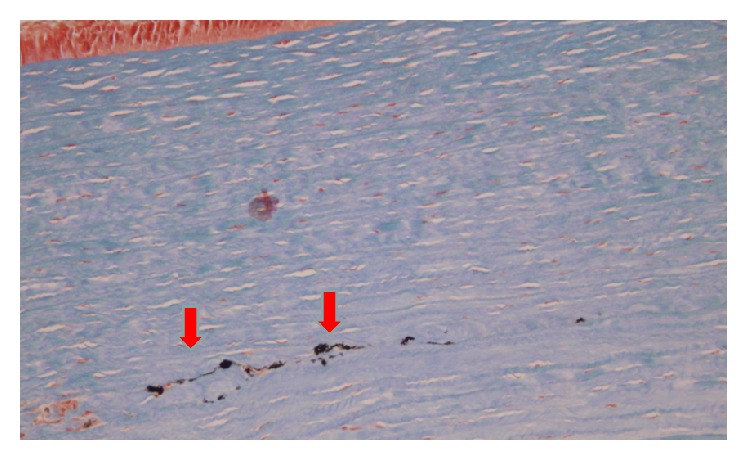
Corneal stroma sample in horizontal orientation stained with Masson trichrome. Solid red arrows showing the CNTs within the corneal tissue.

**Figure 6:**
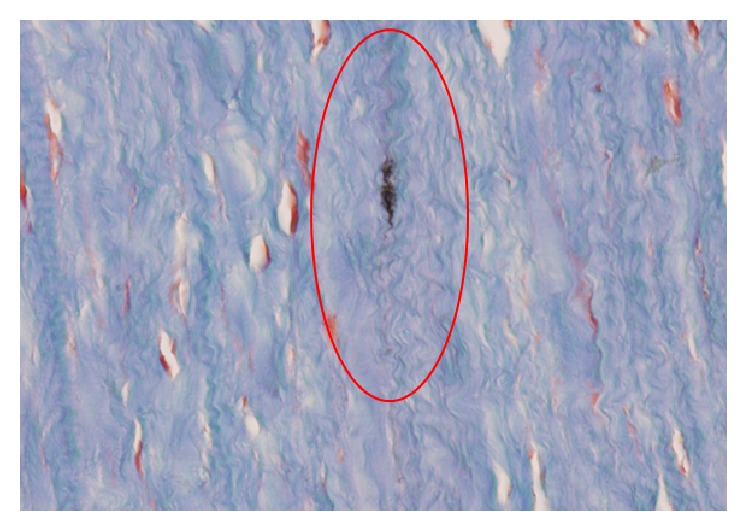
Corneal stroma sample vertically oriented with Masson trichrome staining. Red circle showing the strong adhesion of the tissue around the CNTs.

Figure 4 shows a sample of corneal stroma, where the collagen fibers are oriented vertically and stained with blue Alcian to illustrate that there are no fibrous scaring and no alterations in the mucopolysaccharides of the corneal stroma. It also shows that there are no signs of active inflammation after the procedure. The latest is confirmed when the Masson trichrome staining is performed (Figure 5: solid red arrows showing the carbon nanotubes within the corneal tissue).  Figure 6 illustrates another sample, again vertically oriented, stained with Masson trichrome to show that there is no inflammation and no foreign body giant cell reaction against the CNTs implanted. It also shows a stronger adhesion of the tissue in the area surrounding the CNTs. Other interesting findings that were observed in the pathology report were the absence of neovascularization and that there was no apoptosis of the keratocytes on the samples examined.

### 3.3. Biomechanical Evaluation

**Figure 7:**
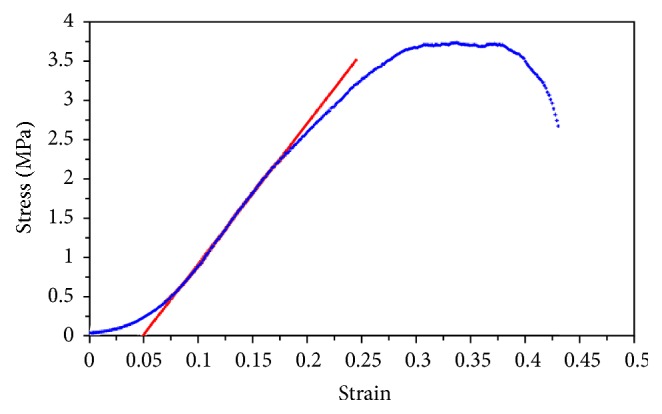
Typical stress-strain curve. Blue points represent the experimental data, and red straight line, the linear fitting used to obtain Young's modulus.

Figure 7 shows a typical stress-strain curve, where several regions can be observed. On one hand, the so-called toe region can be observed, typical in most soft tissues, where the relation between stress and strain is not linear, and the slope increases with stress. On the other hand, the elastic region can be observed, where the relation between the stress and the strain is almost linear. In this region, Young's modulus is calculated as the slope of the straight line (red line in Figure 7). When the stress is increased above the elastic region, the shape of the curve changes and the plastic regions begin. In this region the tissue suffers nonreversible changes. The tissue is not able to recover its original shape in case the force is removed. Finally, the breakdown point of the tissue is reached [21].

Table 1 shows Young's modulus, yield stress, and failure strain obtained for each one of the samples. It can be seen from Young's modulus that there was a trend towards a higher rigidity for the Reference 2 sample (CNTs 1 mg/mL), although this difference was not statistically significant (*p* > 0.05). The yield stress increased for References 1 and 2 compared to the Control sample. However, this difference was only statistically significant for Reference 1 (*p* < 0.05). Also, for Reference 2 there was an increase of the failure strain, but again it was not statistically significant (*p* > 0.05).

## 4. Discussion

The present study evaluates the safety and biocompatibility of carbon nanotubes (CNTs) in ocular tissues. It also assesses the capability of the nanomaterials to increase the biomechanical properties in the corneal stroma. To the best of our knowledge, this is the first scientific investigation that evaluates the behaviour of carbon nanomaterials in ocular tissues.

The unique characteristics of CNTs have kept the attention in many areas within the scientific arena, such as electronics, physics, and mechanics. Also, in nanomedicine the interest for these materials has significantly grown during the last decade as advances in technology allow isolating and developing pure forms of carbon nanostructures. As nanotechnology is defined by the size of a material (generally 1–100 nm) or manipulation on the molecular level, it involves a broad range of nanoscaled materials used in various fields of biology and medicine [22]. Nanotechnology offers promising perspectives in biomedical research as well as in clinical practice. The high versatility of carbon nanomaterials brings many opportunities as therapeutic agents in the field of nanomedicine. Among the different interesting and unique properties that characterized carbon nanostructures, its biocompatibility, optical transparency, and mechanical properties make these structures an excellent option to develop new alternatives of personalized medicine and treat several pathologies in the ophthalmology field.

Corneal ectatic disorders are characterized by a progressive corneal thinning that induces alterations in the morphology of the cornea, which negatively impacts the patient's visual function. The mechanical stability of the cornea is primarily determined by the structure of the collagen molecules and their spatial arrangement, which is orthogonal in normal patients. However, the equilibrium of this architecture is lost in eyes with ectatic corneal pathologies, which leads to biomechanical alterations that are responsible for the instability and weakness of the tissue in these diseases [3]. To this day, the only surgical technique that has demonstrated to stabilize and stop the progressive nature of these disorders is riboflavin UV-A light corneal collagen cross-linking (CXL) [12]. This procedure increases the stiffness of the cornea by a photo oxidative reaction that creates covalent bonds between the collagen fibers of the corneal tissue [12]. However, it is a very long surgical procedure, which makes it uncomfortable for both the surgeon and the patient. Additionally, because of the phototoxic effect of the UV light, it cannot be performed in thin corneas, which is characteristic in ectatic disorders and may present some complications related to the epithelial debridement as well as alterations in the transparency of the tissue. Therefore, using carbon nanomaterials in order to reinforce the corneal stroma will have several advantages as an alternative therapeutic option for such pathological conditions due to the excellent mechanical properties that characterized these nanostructures and also because most of the limitations related to the current treatment options can be avoided.

In the present investigation, biocompatibility of CNTs in ocular tissues was assessed by means of histopathology evaluation. It was found that carbon nanomaterials do not induce any inflammatory or foreign body reaction in the corneal stroma. Biocompatibility of CNTs has been previously reported in other fields of biology and medicine, for example, tissue engineering, regenerative medicine, drug delivery systems, and reinforcement of biological tissues [22–24]. Zhao et al. specifically evaluated the cytocompatibility and hemocompatibility of CNTs by cell adhesion assays and hemolytic rate experiments [23]. They concluded that CNTs did not interfere with the viability, metabolic activity, morphology, and spreading of either of the cell types analyzed. In the same way, Koyama and colleagues observed low toxicity and stable (almost inert) biological response by analyzing the systematic study of T-cells in peripheral blood in in vivo experimental animals [24]. In another study, Tan et al. evaluated the biocompatibility of CNTs composites by seeding these nanomaterials together with pulmonary arterial endothelial cells. Results from this study demonstrated high levels of cell viability and adhesion, thus showing excellent biocompatibility of the CNTs [17]. In addition, there are several authors that have analyzed the biological response of these materials in different types of cells and they have found that CNTs are biologically inert and do not show toxicity on the tissues that were evaluated and also that these nanomaterials are cleared by circulation when injected intravenously [25–29].

In the current study, the mechanical properties of the cornea after being implanted with CNTs were also evaluated. The module of elasticity was assessed by means of stress-strain measurements in order to determine which of the samples under analysis showed the most rigid behaviour. Even though a trend towards stiffer results in those samples treated with the higher concentration of CNTs was found, the differences were not statistically significant when compared with those samples untreated. Therefore, if we want to introduce a reinforced material to another material, it is clear that an interaction must exist between the materials involved.

**Figure 8:**
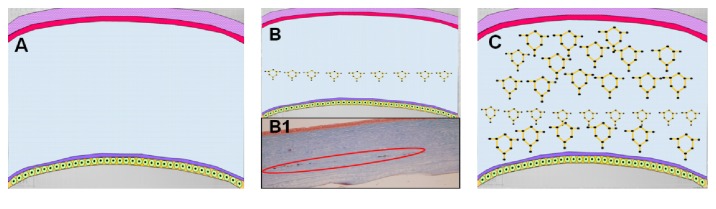
Schematic representation of a cross section of the cornea. (A) Cornea without carbon nanostructures. (B) Cornea with carbon nanostructures in the area where the pocket was created as it was performed in the current study. (B1) Histopathology sample stained with Masson trichrome corresponding to one of the corneas analyzed in the present work where carbon nanostructures can be found just in the area where the stromal pocket was created (red circle). (C) Schematic representation of the cross section of the cornea with an increased distribution of carbon nanomaterials.

In the present study, interactions between the CNTs and the collagen fibers of the corneal stroma were not assessed. Nevertheless, there are some investigations that have demonstrated the adsorption and interaction that is present when combining carbon nanomaterials and collagen fibers by means of theoretical models. Using molecular dynamics simulation, Cazorla characterized the quantum interaction that exists between carbon based nanostructures and collagen-like peptides [30]. In addition, other authors have reported the interaction and adsorption that is present between collagen fibers and carbon nanomaterials by using finite elements analysis and density functional theory [31]. In the same way, aggregation between the collagen fibers and the CNTs has also been demonstrated by measuring the turbidity of the suspension [32]. There are other investigations in which qualitative and chemical assessment have been carried out in order to evaluate the interaction between collagen fibers and carbon nanostructures. Akasaka and col. evaluated the interaction between CNTs and collagen using scanning electron microscopy (SEM) [33]. The authors concluded that CNTs interact strongly and blend with collagen fibers. In the same way, Tosun and McFetridge performed qualitative analysis using SEM and also found an increase in adsorption between the single wall carbon nanotubes and collagen hydrogels [34]. Moreover, this interaction between CNTs and collagen fiber has also been studied using a chemical approach. In the aforementioned study performed by Tan and colleagues, the relationship between CNTs and collagen fibers was also analyzed by spectrometry analysis [17]. Additionally, Cao et al. also evaluated the chemical interaction by means of Fourier transform infrared spectroscopy and they specifically observed that the carboxylic and hydroxyl groups present on the surface of CNTs form covalent and hydrogen bonds with the amino groups of the collagen fibers [16]. As we can see, there is enough scientific evidence published in the literature that has demonstrated the interaction that exists between the CNTs and the collagen by means of theoretical, qualitative, and quantitative assessment. Finally, some of these reports observed that this interaction leads to an increase of the mechanical properties in the collagen. Cao et al. specifically found an increase of the static tensile modulus and strength of the collagen with the addition of CNTs [16]. Tosun and McFetridge also reported that the collagen gels treated with CNTs displayed a significant increase in material stiffness when evaluated by unconfined compression analysis [34]. Nevertheless, as previously mentioned, in the current investigation, even when we observed a trend towards increasing the mechanical properties of the samples treated with CNTs, these changes were not statistically significant. There are some variables, like the small sample under analysis and the methodology used in order to evaluate the mechanical properties, which could have been related to this behaviour. However, we consider that the most important factor that could explain the limited enhancement of the mechanical properties of the samples in our study is that CNTs were inducing their effect on just a small area of the cornea, specifically where the pocket was created (Figure 8). Hence, it is our hypothesis that it is necessary to obtain a more homogeneous distribution of the carbon nanomaterials all over the stroma in order to achieve a significant increase of the mechanical properties in the cornea (Figure 8).

Optimized alternatives including other types of carbon nanomaterials, such as graphene, incorporating drug delivery systems to enhance the penetration and distribution of carbon nanomaterials along the corneal stroma and increasing their chemical properties via functionalization are now being developed by our research group. This will certainly improve the biocompatibility, the optical transparency, and the capacity of these materials to conjugate and interact with other biomolecules, such as corneal collagen fibers.

## 5. Conclusions

In conclusion, histopathology evaluation shows the biotolerance and biocompatibility of carbon nanostructures after being implanted in the corneal stroma. The corneal biomechanical evaluation, as performed in this investigation, shows that there is a trend to obtain more rigidity of the corneal tissue after carbon nanostructures implantation, although these changes were not statistically significant. To the best of our knowledge, this is the first study that aims to assess the biocompatibility and biomechanical properties of the cornea after being implanted with carbon nanomaterials. Thus, further research is necessary in order to comprehend and improve the biomechanical assessment of the present investigation and also to understand the potentials use of these materials and this novel technology for the development of new applications of nanomedicine in visual sciences.

## Figures and Tables

**Table 1:** Number of sample data sets (*n*), Young's modulus, yield stress, and failure strain obtained in each one of the samples.

Group	*n*	Young's modulus (MPa)	Yield stress (MPa)	Failure strain
Control	5	12.1 ± 2.5	1.4 ± 0.3	0.40 ± 0.06
Pocket	6	12.0 ± 4.2	1.9 ± 1.0	0.39 ± 0.08
Reference 1	5	11.8 ± 1.3	2.3 ± 0.3	0.47 ± 0.05
Reference 2	7	13.0 ± 3.9	1.9 ± 0.6	0.41 ± 0.07
